# Investigation of CCL18 and A1AT as potential urinary biomarkers for bladder cancer detection

**DOI:** 10.1186/1471-2490-13-42

**Published:** 2013-09-05

**Authors:** Makito Miyake, Shanti Ross, Adrienne Lawton, Myron Chang, Yunfeng Dai, Lourdes Mengual, Antonio Alcaraz, Evan Gomes Giacoia, Steve Goodison, Charles J Rosser

**Affiliations:** 1 Cancer Research Institute, Orlando Health, Orlando, FL 32827, USA; 2 Department of Pathology, Orlando Health, Orlando, FL 32806, USA; 3 Department of Biostatistics, University of Florida, Gainesville, FL 32601, USA; 4 Laboratory and Department of Urology, Hospital Clínic, Universitat de Barcelona, Barcelona, Spain; 5 Nonagen Bioscience Corporation, Orlando, FL 32827, USA; 6 Section of Urologic Oncology, 1400 S. Orange Ave., Orlando, FL 32806, USA

**Keywords:** Biomarkers, Bladder cancer, Specificity, Urine

## Abstract

**Background:**

In this study, we further investigated the association of two biomarkers, CCL18 and A1AT, with bladder cancer (BCa) and evaluated the influence of potentially confounding factors in an experimental model.

**Methods:**

In a cohort of 308 subjects (102 with BCa), urinary concentrations of CCL18 and A1AT were assessed by enzyme-linked immunosorbent assay (ELISA). In an experimental model, benign or cancerous cells, in addition to blood, were added to urines from healthy controls and analyzed by ELISA. Lastly, immunohistochemical staining for CCL18 and A1AT in human bladder tumors was performed.

**Results:**

Median urinary protein concentrations of CCL18 (52.84 pg/ml *vs.* 11.13 pg/ml, *p* < 0.0001) and A1AT (606.4 ng/ml *vs.* 120.0 ng/ml, *p* < 0.0001) were significantly elevated in BCa subjects compared to controls. Furthermore, the addition of whole blood to pooled normal urine resulted in a significant increase in both CCL18 and A1AT. IHC staining of bladder tumors revealed CCL18 immunoreactivity in inflammatory cells only, and there was no significant increase in these immunoreactive cells within benign and cancerous tissue and no association with BCa grade nor stage was noted. A1AT immunoreactivity was observed in the cytoplasm of epithelia cells and intensity of immunostaining increased with tumor grade, but not tumor stage.

**Conclusions:**

Further development of A1AT as a diagnostic biomarker for BCa is warranted.

## Background

Non-invasive urine tests for the early detection or post-surgical surveillance of bladder cancer (BCa) are highly desirable for both the patient and the healthcare system. Currently, voided urinary cytology (VUC) is the most widely used non-invasive urine test, with reported specificities ranging from 85-100% and sensitivities ranging from 13-75% [1,2]. Two single-protein biomarker urine-based assays, bladder tumor antigen (BTA) test and nuclear matrix protein-22 (NMP-22) test, have been developed and FDA approved for use in this context. However, these assays have significant limitations. The BTA tests (BTA stat™ and BTA TRAK™ (Polymedco Inc. Cortlandt Manor, NY, USA) have diagnostic sensitivities ranging from 29-83% and specificities ranging from 56-86% [3,4]. In addition, we and others have demonstrated in an experimental model that hematuria adversely affects the accuracy of the BTA assay [5,6]. The NMP-22 tests (NMP22® Bladder Cancer ELISA Test Kit and the NMP22® BladderChek® point-of-care test, Alere Scarborough, Inc. Waltham, MA) have diagnostic sensitivities ranging from 47-100% and specificities ranging from 55-98% [7,8]. Atsu *et al.* and others have recently demonstrated in an experimental model that NMP-22 assays measure the cellularity or amount of cell turnover that may be introduced into the urine by a variety of conditions, including hematuria, infection and instrumentation [9,10]. Thus, the search for more accurate urine-based biomarkers continues.

Through genomic and proteomic profiling of urine components, we have previously identified a panel of biomarkers that can outperform current urine-based biomarkers for the non-invasive detection of BCa [11-14]. In a case-controlled validation study, the urinary concentrations of our panel of 14 biomarkers (IL-8, MMP-9, MMP-10, SDC1, CCL18, PAI-1, CD44, VEGF, ANG, CA9, A1AT, SPP1, PTX3, and APOE) were measured by enzyme-linked immunosorbent assay (ELISA) in voided urines from 127 patients (64 tumor bearing subjects) [15-18]. Of these 14 biomarkers, two biomarkers (CCL18 and A1AT) had high correlation coefficients (Spearman correlation coefficient >0.76) with urinary blood content and therefore, rather than measuring a valid tumor antigen the biomarker may be merely a surrogate for hematuria. Subsequently, these two biomarkers have been excluded from ongoing multiplex studies [19] until we can clarify the source of these protein biomarkers. Herein, we report the urinary concentrations of CCL18 and A1AT in an independent larger case–control study, and illustrate in an experimental model the influence of cellular proteins and whole blood on the performance of these potential urine-based biomarkers.

## Methods

### Ethics statement

Under Institutional Review Board approval by the committees at MD Anderson Cancer Center Orlando and Hospital Clinic of Barcelona, written informed consent was obtained prior to collection and storage of biological specimens (voided urine samples and blood) in genitourinary biorepositories. Furthermore under Institutional Review Board approval by the committee at MD Anderson Cancer Center Orlando with a waiver of written informed consent, archived bladder tissues from the Department of Pathology at Orlando Health was identified for immunohistochemical analysis. The above review boards monitored study recruitment and study compliance.

### Patients and data collection

For the urinary ELISA validation study, 308 non-consecutive subjects (102 with BCa) from MD Anderson Cancer Center Orlando and Hospital Clínic of Barcelona were available for analysis. The control cohort consisted of 206 individuals (47 with voiding symptoms, 44 with urolithiasis, 9 with gross hematuria, 14 with urinary tract infection and 92 without any diagnosed condition). Patients with a history of renal dysfunction were excluded. The cohort of 308 subjects served as our phase II (validation study) according to the International Consensus Panel on Bladder Tumor Markers and findings were reported according to the STARD criteria [20]. For the experimental model, three healthy volunteers (2 males, 1 female, mean age 36 years) provided urine and blood samples. For the immunohistochemical study, formalin-fixed paraffin embedded blocks containing 165 bladder tumor tissue specimens and 8 benign tissue specimens were retrieved from the Orlando Health Department of Pathology.

### Specimen processing

Fifty milliliters of voided urine from each subject was assigned a unique identifying number before delivery to the laboratory for processing. Each urine sample was centrifuged at 1000 × *g* 4°C for 10 min. The supernatant was decanted and aliquoted, and the urinary pellet was snap frozen. Both the supernatant and pellet were stored at -80°C prior to analysis. Urine supernatant protein concentration was determined using Pierce 660-nm Protein Assay Kit (Thermo Fisher Scientific Inc., Waltham, MA, USA). Patients with significant proteinuria were excluded.

### Enzyme-linked immunosorbent assays for urinary CCL18 and A1AT

The levels of human CCL18 (Cat # ab100620, Abcam, Cambridge, MA) and human A1AT (Cat# ab108799, Abcam) in urine samples were monitored using ELISA. The assays were conducted according to the manufacturer’s instructions. Laboratory personnel were blinded to final diagnosis. Calibration curves were prepared using purified standards for each protein assessed. Curve fitting was accomplished by either linear or four-parameter logistic regression following manufacturer’s instructions. Urinary creatinine levels were monitored with a commercial ELISA assay (Cat# KGE005 R&D Systems Inc., Minneapolis, MN, USA) as previously described [21].

### Cell lines and culture

Human bladder cancer cell lines T24 (ATCC, Manassas, VA) and UM-UC-14 (a generous gift from Dr. H. Bart Grossman, The University of Texas M.D. Anderson Cancer Center, Houston, TX) [22] were available for analysis. The benign human bladder cell line, UROtsa, was a generous gift from Dr. Donald Sens at the University of North Dakota School Of Medicine (Grand Forks, ND) [23]. T24 and UM-UC-14 cell lines were maintained in RPMI 1640 media. UROtsa cells were maintained in McCoy’s 5A medium (Life Technologies, Inc., Gaithersburg, MD). All media were supplemented with 10% fetal bovine serum, 100 units/ml of penicillin and 100 μg/ml of streptomycin. All cells were incubated at 37°C in a humidified atmosphere of 5% CO_2_.

### Experimental model

**Figure 1:**
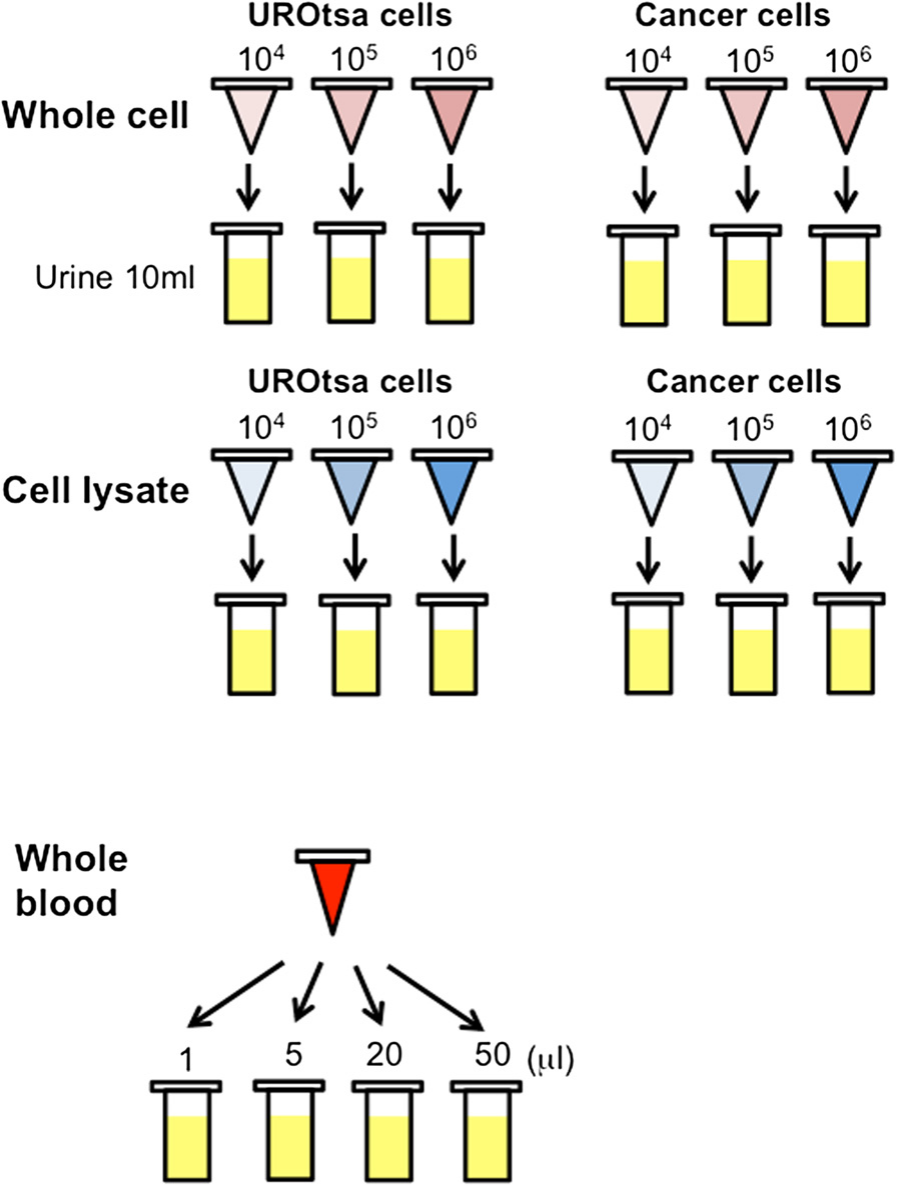
**Schematic of the experimental model.** Low concentration (1×10^4^), medium concentration (1×10^5^) and high concentrations (1×10^6^) of intact UROtsa benign human bladder cells, or a mixture of human bladder cancer lines, T24 and UM-UC-14 were added to 10 ml of pooled urine from three healthy controls. Low protein concentration of cellular lysate (4 μg), medium concentration (40 μg) and high concentrations (400 μg) of UROtsa benign human bladder cells, or from a mixture of human bladder cancer lines, T24 and UM-UC-14 were added to 10 ml of pooled urine from three healthy controls. Whole blood (1, 5, 20 and 50 μl) was also added to 10 ml of pooled urine from healthy controls.

The experimental model was essentially as previously published [6,10]. Figure 1 illustrates the experimental model components and dilutions. Briefly, 10 milliliters of whole blood in heparinized tube and 200 ml of freshly voided urine samples in sterile containers were obtained from three healthy controls. The urine samples from the three healthy subjects were pooled, mixed and distributed into 10 ml aliquots in 15 ml centrifuge tubes. The human bladder cell lines were washed, trypsinized and counted. For UROtsa, 1×10^4^ cells (low concentration), 1×10^5^ cells (medium concentration) and 1×10^6^ cells (high concentration) cells were each added to 10 ml pooled urine samples in triplicate. Equal numbers of T24 and UM-UC-14 were pooled, and 1×10^4^ pooled cells (low concentration), 1×10^5^ pooled cells (medium concentration) and 1×10^6^ pooled cells (high concentration) cells were added to 10 ml of pooled urine samples in triplicate. For cell lysate analyses, 1×10^6^ cells from each cell line were lysed with RIPA buffer (Pierce, Rockford, IL) and total protein concentration measured. The total protein extracted from 1×10^6^ cells of UROtsa, T24 and UM-UC-14 were 431 μg, 471 μg, 280 μg and 420 μg, respectively, with a mean total protein extract of 400 μg. In the spiking experiments, 4 μg, 40 μg and 400 μg of cellular proteins from either UROtsa or the pooled BCa cell lines were used, corresponding to ~1×10^4^ cells (low concentration), ~1×10^5^ cells (medium concentration) and ~1×10^6^ cells (high concentration). UROtsa lysates and pooled cancer cell lysates were added to pooled urine samples in triplicate. To monitor the influence of hematuria, pooled whole blood from three healthy subjects was added in triplicate to 10 ml of pooled urine samples in the following amounts; 1 μl, 1/10000 final dilution; 5 μl, 1/2000 final dilution; 20 μl, 1/500 final dilution; 50 μl, 1/200 final dilution and zero control. The number of red blood cells (RBC) in each urine sample was determined by microscopic examination before and after adding whole blood. Standard urinalysis was performed with MULTISTIX PRO Reagent Strips (Bayer HealthCare, Elkhart, IN).

### Immunohistochemistry

A total of 173 paraffin blocks were verified histologically by H&E staining. For immunochemical staining, blocks were cut in 5 μm sections and placed on a Superfrost Plus Miscroslide. Sections were deparaffinized, followed by antigen retrieval using citric acid buffer (pH 6.0, 95°C for 20 min). The slides were treated with 1% hydrogen peroxide in methanol to block endogenous peroxidase activity. After 20 min blocking in 1% bovine serum albumin (BSA), the slides were incubated overnight at 4°C with anti-human CCL18 antibody (MAB394; mouse monoclonal, dilution 1/500 in 1% BSA) from R&D Systems Inc., or anti-human A1AT antibody (NBP1-90309; rabbit polyclonal, dilution 1/2500 in 1% BSA) from Novus Biologicals Inc. (Littleton, CO). Next, the slides were incubated with 2 μg/mL of biotinylated anti-mouse or anti-rabbit IgG secondary antibody (Vector Laboratories, Burlingame, CA) for 30 min at room temperature. Subsequently, the sections were stained using Standard Ultra-Sensitive ABC Peroxidase Staining kit (Pierce/Thermo Fisher Scientific, San Jose, CA) and 3, 3'- diaminobenzidine (DAB; Vector Laboratories), counterstained by hematoxylin, dehydrated, and mounted with a cover slide. Human liver, known to stain strongly for CCL18 and A1AT, was used as a positive control, and negative controls were performed by omitting the primary antibodies. Using light microscopy, two investigators (MM and AL) interpreted immunostaining results blinded to specimen and patient data. A third investigator (CJR) reviewed discrepancies and rendered a final score. The location of immunoreactivity (*e.g.,* nuclear, cytoplasm, cell membrane, and stroma) was noted. CCL18 immunostaining was positive only in inflammatory cells in the stroma. Three randomly chosen high power fields (1 HPF = 0.237 mm^2^) were analyzed for CCL18-positive cells in the stromal area and averaged in each case. A1AT immunostaining was positive only in the cytoplasm of epithelial cells. Immunostaining intensity was reported as weak, moderate or strong.

### Data analysis

The Wilcoxon rank sum test was used on ELISA data to determine the association between urinary CCL18, A1AT and BCa status. Nonparametric receiver operating characteristic (ROC) curves were plotted and the ability of the biomarker to predict the presence of BCa was estimated by calculating the area under the ROC curves (AUROC). The sensitivity and specificity of the biomarker at the optimal cutoff value was defined by calculating the Youden index [24]. Comparison of immunohistochemical distribution data was performed using Chi square test. Spearman rank correlation coefficients were used to examine the correlation between urinary CCL18 and A1AT concentrations and urinary hemoglobin concentration. The association between CCL18 and A1AT levels and BCa was tested using the Mann Whitney test. Statistical significance in this study was set at *p* < 0.05 and all reported *p* values were 2-sided. All analyses were performed using PRISM software version 5.00 (San Diego, CA).

## Results

### CCL18 and A1AT in voided urine samples

**Figure 2:**
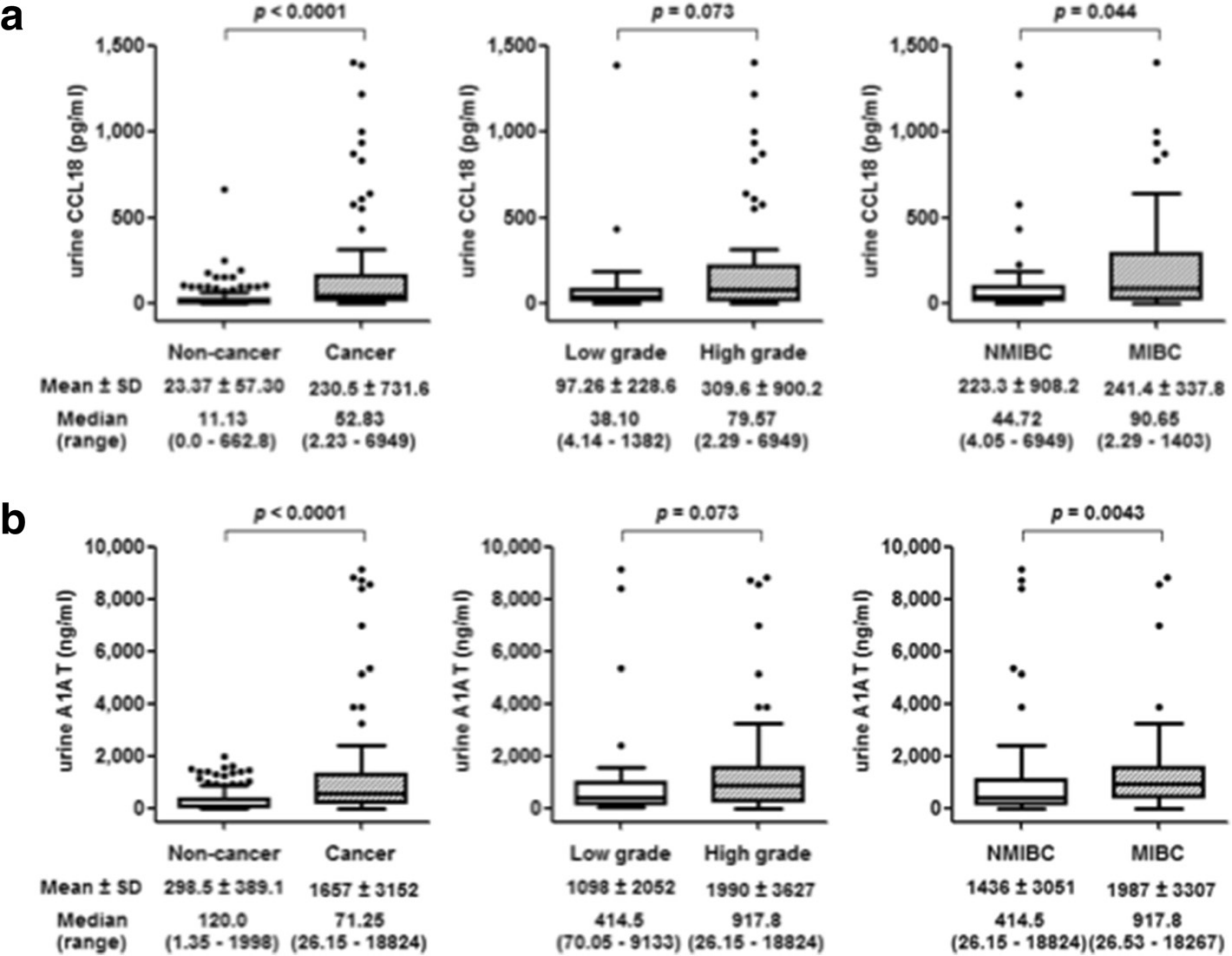
**Urinary biomarker levels. a)** Comparison of urine concentrations of CCL-18 between the cancer and non-cancer groups (left panel), low-grade and high-grade (middle panel) and non-muscle invasive bladder cancer (NMIBC) and muscle-invasive bladder cancer (MIBC). **b)** Comparison of urine concentrations of A1AT between the same groups. Median levels are depicted by horizontal lines within boxes, standard deviations are depicted by bars. Significance (*p < 0.05*) was assessed by the Wilcoxon rank sum test.

**Figure 3:**
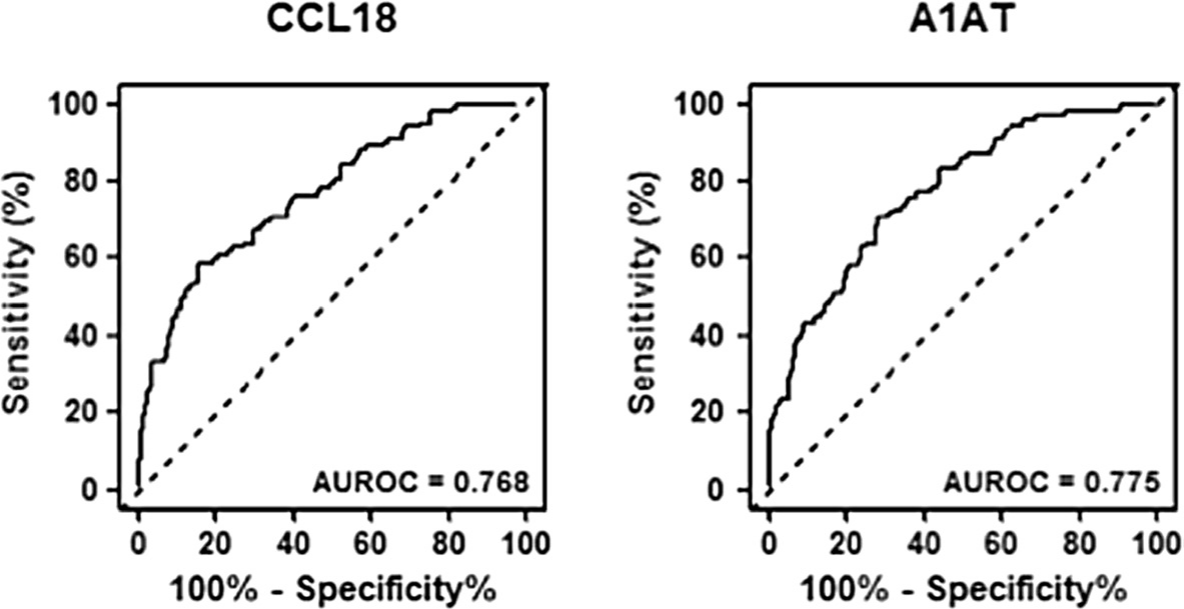
**Diagnostic performance of urinary CCL18 and A1AT.** Receiver operator characteristic (ROC) curves were calculated from the analysis of urine samples obtained from a cohort of 308 subjects (102 with confirmed bladder cancer) for CCL18 and A1AT. AUROC, area under the ROC curve.

Table 1 depicts demographics and clinical characteristics of the study cohorts. Ninety percent of the BCa subjects sampled were Caucasian (median age 69 years) with 60% noted to have non-muscle invasive bladder cancer (NMIBC) and 37% with low-grade disease. In the cancer cohort, urinary cytology achieved a diagnostic sensitivity of 39%. Median urinary protein concentrations of CCL18 (52.84 pg/ml *vs.* 11.13 pg/ml, *p* < 0.0001) and A1AT (606.4 ng/ml *vs.* 120 ng/ml, *p* < 0.0001) were significantly elevated in BCa subjects compared to controls. Furthermore, median urinary CCL18 was significantly elevated in muscle invasive bladder cancer (MIBC) compared to NMIBC (90.65 pg/ml *vs*. 44.72 pg/ml, *p* = 0.044), and approached significance (Figure 2a) in high-grade compared to low-grade disease (79.57 pg/ml *vs.* 38.10 pg/ml, *p* = 0.073). Similarly, median urinary A1AT was significantly elevated in MIBC compared to NMIBC (978.1 ng/ml *vs.* 414.5 ng/ml, *p* = 0.0042) and approached significance (Figure 2b) in high-grade compared to low-grade (917.8 ng/ml *vs.* 414.5 ng/ml, *p* = 0.073). The ability of the tested biomarkers to predict the presence of BCa was analyzed using nonparametric ROC analyses, according to National Cancer Institute guidelines [25]. Based on the AUROC, we determined Youden index cutoff values to maximize the sum of sensitivity and specificity. Urinary CCL18 had an area under the curve of 0.768 (95% CI: 0.713-0.824) (Figure 3), achieved a sensitivity of 70.4%, specificity of 67.7%, positive predictive value of 53.1% and a negative predictive value of 81.5%. Urinary A1AT had an area under the curve of 0.775 (95% CI: 0.721-0.829) (Figure 3), achieved a sensitivity of 70.6%, specificity of 71.8%, positive predictive value of 55.4% and negative predictive value of 83.2%.

**Table 1:** Demographic and clinicopathologic characteristics of 308 subjects comprising ELISA study cohort and 173 subjects comprising IHC study cohort

	**ELISA Cohort**		**IHC Cohort**	
	**BCa (%) n = 102**	**Controls (%) n = 206**	**BCa (%) n = 165**	**Controls (%) n = 8**
**Median Age (range, y)**	**69 (20 – 93)**	**56 (18 – 89)**	**67 (47 – 91)**	**59 (42 – 83)**
**Male : Female ratio**	**84 : 18**	**152 : 54**	**132 : 33**	**7 : 1**
**Race**				
**White**	**91 (90%)**	**135 (66%)**	**137 (83%)**	**7 (88%)**
**African American**	**5 (5.9%)**	**20 (10%)**	**8 (5%)**	**0 (0%)**
**Other**	**6 (6.1%)**	**51 (24%)**	**20 (12%)**	**1 (12%)**
**Positive FISH**	**40 / 74 (54%)**	**2/22(9%)**	**N/A**	**N/A**
**Suspicious/positive cytology**	**37 / 94 (39%)**	**2/22(9%)**	**N/A**	**N/A**
**Median follow-up (months)**	**14**	**4**	**11**	**3**
**Clinical stage**				
**Tis**	**6 (6%)**		**15 (9%)**	
**Ta**	**41 (40%)**		**15 (9%)**	
**T1**	**14 (14%)**		**15 (9%)**	
**≥T2**	**41 (40%)**		**120 (73%)**	
**Tumor grade**				
**Low**	**38 (37%)**		**36 (22%)**	
**High**	**64 (63%)**		**129 (78%)**	
**Median tumor size (cm)**	**3.0**		**3.5**	
**Median urinary CCL18 pg/ml (range)**	**52.84**	**11.13**		
	**(2–6949)**	**(0–662)**		
**Median urinary A1AT ng/ml (range)**	**606.4**	**120.0**		
	**(26–8830)**	**(1–1997)**		
**Median urinary protein μg/ml (range)**	**24.2**	**38.9**		
	**(24–789)**	**(24–561)**		

### Experimental model

Urine and blood samples were obtained from three healthy volunteer controls for analysis in the experimental model. There was no evidence of gross hematuria, urinary tract infection or any biochemical abnormalities in any volunteer urine samples. Both urinary dipstick and urinary microscopy were negative for hematuria, however, urinary hemoglobin measured by ELISA assay revealed trace amounts in all samples (3.63 ± 0.59 ng/ml). In these healthy controls, the mean urinary CCL18 level was 5.96 ± 7.73 pg/ml, and the mean urinary A1AT level was 1170 ± 71.94 ng/ml (Table 2).

**Table 2:** Results of CCL18 and A1AT in experimental hematuria model

**Dilution (volume of whole blood)**	Red blood cells ( /hpf * )	**Concentration (mean ± SD)**			**Urinary protein (dipstick test)**
		**Hemoglobin (ng/ml)**	**CCL18 (pg/ml)**	**A1AT (ng/ml)**	
0	0 ± 0	3.63 ± 0.59	5.96 ± 7.73	1170.70 ± 71.94	negative
1/10,000 (1 μl)	0.6 ± 1.0	174.28 ± 15.69	7.06 ± 7.78	1227.31 ± 1.22	negative
1/2,000 (5 μl)	2.4 ± 1.2	2289.34 ± 479.07	6.80 ± 9.61	1252.49 ± 2.92	30 mg/dL
1/500 (20 μl)	10.0 ± 4.4	3633.39 ± 2006.87	16.62 ± 22.17	1258.43 ± 13.48	100 mg/dL
1/200 (50 μl)	17.3 ± 3.0	7898.05 ± 184.67	31.90 ± 43.19	1273.16 ± 5.53	300 mg/dL

*hpf high power field.

**Figure 4:**
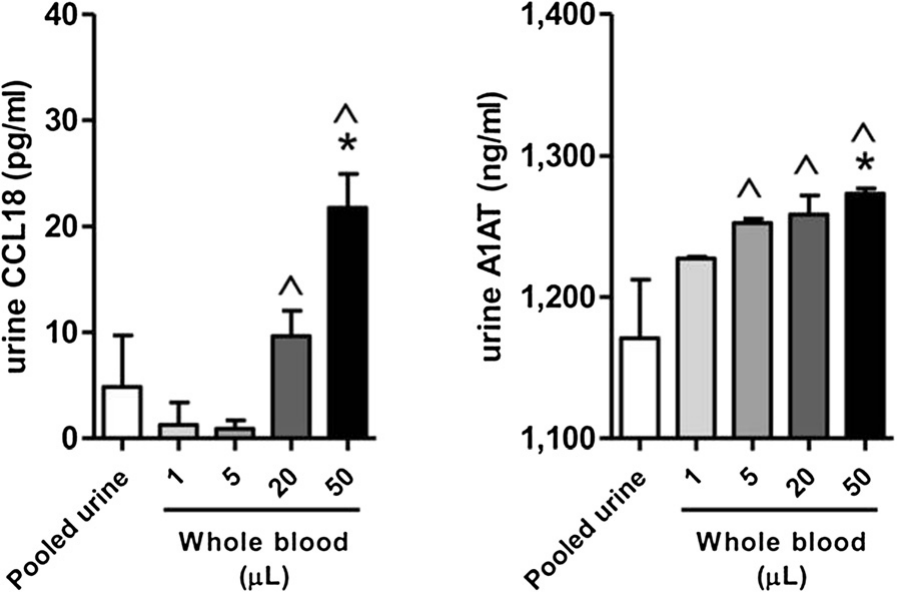
**Analysis of CCL18 and A1AT biomarker performance in an experimental model.** Using the experimental model depicted in Figure 1, urinary levels of CCL18 and A1AT were analyzed by ELISA. The addition of whole blood resulted in an increase in CCL18 as well as an increase in A1AT. Error bars indicate standard deviations. *, significance (*p* < 0.05) compared to pooled urines from healthy subjects. ^, significance (*p* < 0.05) compared to corresponding lower concentration.

Urine samples were pooled, and whole cells or cell lysates of the cancer cell line pool and UROtsa cells were added to the urine sample (as depicted in Figure 1) and re-analyzed for CCL18 and A1AT using ELISA. The addition of a high concentration (400 μg) of protein lysate from UROtsa cells, or medium to high concentration (40 μg to 400 μg) of protein lysate from pooled cancerous cells resulted in a significant increase in test sample CCL18 (Additional file 1). Furthermore, the addition of whole blood (50 μL) resulted in a significant increase in CCL18 (*p* < 0.05) (Figure 4). As for A1AT, the addition of high concentration of whole blood (50 μL) resulted in a significant increase in test sample A1AT levels (*p* < 0.05) (Figure 4).

With the addition of only 1 μl of whole blood to 10 mL of test urines (1/10,000 dilution), the mean urinary hemoglobin level was 174.28 ± 15.69 ng/ml, and microscopy revealed a median 1 RBC/hpf. This level would be termed ‘Negative’ or ‘Trace’ in clinical tests such as the Multistix Pro dipstick test (negative blood is <100ng/ml). At this level, CCL18 and A1AT concentrations were unaffected at 7.06 ± 7.78 ng/ml and 1227.31 ± 1.22 ng/ml, respectively. With the addition of 50 μl of whole blood to 10 mL of urine, all urines had visibly gross hematuria, the mean urinary hemoglobin level was 7,898.05 ± 184.67 ng/ml and median of 17 RBC/hpf was noted. At this level, CCL18 concentration was raised to 31.90 ± 43.19 ng/ml (~4.5 fold increase), but the A1AT concentration was similar to controls at 1,273.16 ± 5.53 ng/ml (Table 2). As the concentration of whole blood added to the urine samples increased, the mean urinary hemoglobin level, the extent of hematuria assessed by microscopy, and the mean urinary concentrations of CCL18 and A1AT increased accordingly (Table 2). There were high correlation coefficients between hemoglobin and CCL18 (Spearman correlation coefficient = 0.90) and hemoglobin and A1AT (Spearman correlation coefficient = 1.00).

### Immunohistochemical analysis of bladder tumors

**Figure 5:**
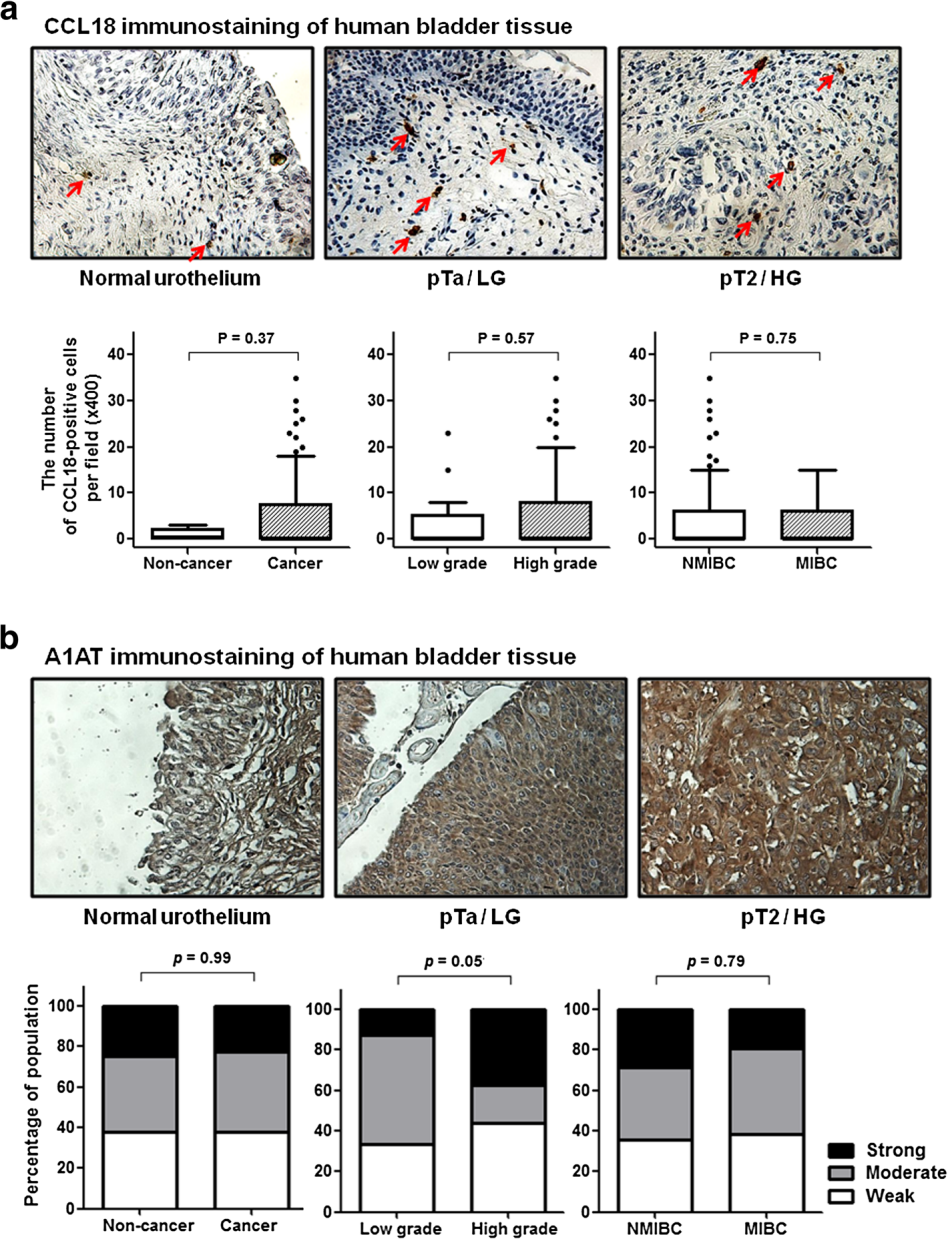
**Assessment of CCL18 and A1AT in bladder tissue. a)** Representative immunostaining of benign bladder for CCL18 (top-left), low-grade non-muscle invasive bladder cancer for CCL18 (top-middle), and high-grade muscle invasive bladder cancer for CCL18 (top-right). Red arrows indicate CCL18-positive cells in the stroma. CCL18 staining was present only in inflammatory cells associated with the stroma. Lower panels are boxplots of CCL18 immunohistochemical staining intensity of benign bladder *vs.* bladder cancer, low-grade *vs.* high-grade, non-muscle invasive bladder cancer (NMIBC) *vs*. muscle invasive bladder cancer (MIBC). Error bars indicate standard deviations. Wilcoxon rank sum test was used to assess significance. **b)** Representative immunostaining of benign bladder for A1AT (top-left), low-grade non-muscle invasive BCa for A1AT (top-middle), and high-grade muscle invasive BCa for A1AT (top-right). A1AT staining was present in the cytoplasm and stroma. A1AT staining varied from weak and focal to strong and diffuse. Lower panel shows column bar graphs of A1AT immunohistochemical staining intensity of benign bladder *vs.* bladder cancer, low-grade *vs.* high-grade, NMIBC *vs.* MIBC.

The study cohort consisted of 8 subjects without cancer and 165 non-consecutive subjects with BCa (37 subjects with low-grade BCa and 128 subjects with high-grade BCa, 45 subjects with NMIBC and 120 MIBC). Immunohistochemical staining patterns for CCL18 and A1AT were assessed in both malignant and normal bladder tissue. No epithelial staining was evident for CCL18, however, inflammatory cells in the stromal were positive. The number of CCL18-positive inflammatory cells per high power field was not increased in bladder tumors compared to controls (1.0 ± 1.2 *vs.* 4.5 ± 6.9, *p* = 0.57). In addition, an increase in CCL18-positive inflammatory cells was not associated with higher grade or higher stage disease (Figure 5a). Immunostaining for A1AT revealed a predominantly epithelial and cytoplasmic localization. Intensity of staining ranged from weak and focal to strong and diffuse. No difference in staining intensity was seen between benign and cancer (*p* = 0.99). Staining intensity increased with an increase in tumor grade (*p* = 0.05), however, staining pattern was not significantly associated with tumor stage (*p* = 0.79) (Figure 5b).

## Discussion and conclusions

We have previously identified CCL18 and A1AT as potential biomarkers for the detection of BCa in voided urine samples [15,18]. CCL18 is a member of the serum-based cytokine family of secreted proteins involved in immunoregulatory and inflammatory processes. CCL18 is thought to promote the invasiveness of cancer cells by triggering integrin clustering and enhancing their adherence to the extracellular matrix, and a receptor (PITPNM3) for this cytokine has been recently identified [26]. CCL18 has been identified in gynecological tumors but not urologic tumors [27,28]. A1AT, also known as SERPINA1, is a member of a family of serine proteases inhibitors. Specifically A1AT irreversibly inhibits trypsin, chymotrypsin and plasminogen activator. Serpins are known to have diverse but critical roles in the cell, including regulation of homeostasis, cellular survival and blood clotting [29]. Within the oncology literature, reports describe genetic aberrations in cancers, elevated levels in the sera of cancer patients, and survival disadvantage in tumors expressing A1AT [30,31].

In our early studies, we noted that these biomarkers had a relatively high correlation (Spearman correlation coefficient > 0.76) with urinary hemoglobin. Given the confounding effects of hematuria that we and others have described for the urine-based BCa detection assays BTA and NMP-22 [6,10], we set out to more methodically analyze the association of CCL18 and A1AT with BCa by analyzing cohorts of urine and tissue samples. In this study, ELISA analysis of urine samples from a cohort of 308 subjects confirmed our previous findings [19] that both CCL18 and A1AT are significantly elevated in the urines of subjects with BCa.

To investigate the potential influence of hematuria and other factors on the performance of these biomarkers we employed an experimental model. Although the model does not mimic the actual physiological situation exactly, it does enable the identification of potential sources of specific analytes and to what extent incursion of blood components into the urine may influence the data. We previously used a similar model approach to demonstrate that BTA urine tests primarily detect a serum-based protein [6], and that NMP-22 urine tests monitor cellular turnover, rather a specific bladder tumor antigen [10].

Analyses from the model and the ELISA assays revealed different characteristics for CCL18 and A1AT with respect to them being potentially reliable BCa diagnostic biomarkers. For CCL18, the first observation is that three healthy control samples had very low urinary CCL18 levels (5.96 pg/ml). The median level of CCL18 in the non-cancer samples from the 308 subject cohort was also very low (23.4 pg/ml). A low baseline level is an advantage that can enable a clear distinction between healthy and disease state for a given assay. Conversely, if the differential between urine and blood is large, then a small amount of hematuria may have a significant impact. In the spiking experiment, we observed that 50 μl of blood in 10 ml of urine, a level that would be termed ‘gross hematuria’ in clinical tests, raised the CCL18 level in healthy controls to 31.9 pg/ml, an increase of ~5.3 fold. In the 308 subject cohort, the median level of CCL18 was 10-fold higher in BCa subjects (230.5 pg/ml *vs.* 23.4 pg/ml). The addition of benign and tumor cell lysates to the urine sample is designed to indicate whether the release of ubiquitous cellular factors may be the source of the biomarker. Increased cellular turnover is to be expected in a malignant condition, and so such factors, for example NMP22, may increase even though they are not actually cancer-specific biomarkers. Due to the low levels of CCL18 in the healthy urine samples, the addition of cell lysates from benign and tumor cells did significantly impact the CCL18 levels. Finally, immunohistochemical analysis of bladder tumor tissues revealed that CCL18 was present only in the inflammatory cells located in the stroma. No difference in the number of the immunoreactive cells was observed in benign versus cancerous tissue, or among the various grades or stages of bladder cancer. Together, these findings suggest that CCL18 monitoring is unlikely to be a reliable biomarker for the non-invasive detection of BCa.

The analysis of the A1AT biomarker revealed opposite characteristics for the most part. ELISA data and the experimental model confirmed that A1AT is present at high levels in healthy and non-cancer subject urine samples. The median level in the 308 subject cohort was 120 ng/ml in non-cancer cases, rising 5.5-fold to 606.4 ng/ml in subjects with confirmed BCa. The median level in the three healthy volunteer samples was intermediate (1,170 ng/ml). Lower levels in the non-cancer subjects from the ELISA data is most likely due to degradation with freezing and storage in these samples compared to the fresh urines obtained from the volunteers. The high baseline level of A1AT in healthy urine samples may not be ideal for diagnostic evaluation, but the impact of hematuria on A1AT assays would be expected to be less pronounced. Accordingly, gross hematuria in the experimental model (50 μl of blood into 10 ml of urine) raised A1AT to 1,273 ng/ml, an increase of only 9%. Compare this to the >400% rise in CCL18 in the same experimental model conditions. The addition of benign or cancer cell lysates in the experimental model had no impact on the A1AT levels. Previous preliminary research has linked the presence of urinary A1AT as a biomarker for renal dysfunction [32]. However we took precautions in our study to minimize renal dysfunction as a confounder by excluding subjects with a history of renal dysfunction as well as exclude subjects with grossly elevated urinary protein levels. Recently, researchers have reported that impaired renal function (*i.e.*, reduced glomerular filtration rate) may adversely affect urinary biomarkers performance [33,34]. This is an excellent point and should be taken into consideration in future studies. However, we must stress that we confirmed these A1AT ELISA results by performing immunohistochemistry and thus demonstrated that A1AT is present within urothelial cells. We reported A1AT reactivity was epithelial in normal bladder tissue, and strongly positive in all tumor cells, specifically high-grade cells. Although A1AT IHC may not be particularly useful in differential histological evaluations, it does suggest that the source of the increased A1AT observed in BCa samples is most likely bladder tumor cells. The release into the urine may be via secretion or the turnover of tumor cells at the urine interface. Thus, even though normal urinary levels of A1AT are relatively high, measurement of this biomarker in the context of cancer detection may be worthwhile. The good separation between non-cancer and BCa urinary levels and the limited influence by secondary sources suggest that valid diagnostic cut-off thresholds may be possible for urinary A1AT monitoring.

When novel urinary biomarkers are proposed, the investigation of reliability in the face of potentially confounding effects is warranted, especially those introduced into the urine through bleeding, a common presenting factor in bladder tumor-bearing patients. Further studies into the utility of A1AT as a biomarker for the non-invasive detection of BCa are currently underway.

## Competing interests

Charles J. Rosser and Steve Goodison are officers for Nonagen Bioscience Corporation. Makito Miyake, Shanti Ross, Adrienne Lawton, Myron Chang, Yunfeng Dai, Lourdes Mengual, Antonio Alcaraz, Evan Gomes Giacoia have no COI.

## Authors’ contributions

MM: Acquisition of data. SR: Acquisition of data. AL: Acquisition of data. MC: Statistical analysis. YD: Statistical analysis. LM: Clinical samples, drafting of manuscript. AA: Clinical samples, drafting of manuscript. EGG: Acquisition of data. SG: Study concept and design, drafting of manuscript. CJ: concept and design, drafting of manuscript, funding. All authors read and approved the final manuscript.

## Pre-publication history

The pre-publication history for this paper can be accessed here:

http://www.biomedcentral.com/1471-2490/13/42/prepub

## Supplementary Material

Additional file 1 **Analysis of CCL18 and A1AT biomarker performance in an experimental model.** Using the experimental model depicted in Figure 1, urinary levels of CCL18 and A1AT were analyzed by ELISA. The addition of high concentration of benign cell lysate or medium to high concentration of cancer cell lysate resulted in an increase in CCL18. The addition of cells or cell lysate did not alter A1AT levels. Error bars indicate standard deviations. *, significance (*p* < 0.05) compared to pooled urines from healthy subjects. ^, significance (*p* < 0.05) compared to corresponding lower concentration. Click here for file

## References

[B1] Rife CC Farrow GM Utz DC Urine cytology of transitional cell neoplasms Urol Clin North Am 1979 6 599 612 505675

[B2] Cajulis RS Haines GK 3rd Frias-Hidvegi D McVary K Bacus JW Cytology, flow cytometry, image analysis, and interphase cytogenetics by fluorescence in situ hybridization in the diagnosis of transitional cell carcinoma in bladder washes: a comparative study Diagn Cytopathol 1995 13 214 23 discussion 24 10.1002/dc.2840130307 8575280

[B3] Heicappell R Wettig IC Schostak M Quantitative detection of human complement factor H-related protein in transitional cell carcinoma of the urinary bladder Eur Urol 1999 35 1 81 7 10.1159/000019822 9933798

[B4] Kinders R Jones T Root R Complement factor H or a related protein is a marker for transitional cell cancer of the bladder Clin Cancer Res 1998 4 10 2511 20 9796985

[B5] Oge O Kozaci D Gemalmaz H The BTA stat test is nonspecific for hematuria: an experimental hematuria model J Urol 2002 167 3 1318 9 discussion 1319–20 10.1016/S0022-5347(05)65290-1 11832722

[B6] Miyake M Goodison S Rizwani W Ross S Bart Grossman H Rosser CJ Urinary BTA: indicator of bladder cancer or of hematuria World J Urol 2012 30 6 869 73 10.1007/s00345-012-0935-9 22932760 PMC3537326

[B7] Grossman HB Messing E Soloway M Tomera K Katz G Berger Y Shen Y Detection of bladder cancer using a point-of-care proteomic assay JAMA 2005 293 7 810 6 10.1001/jama.293.7.810 15713770

[B8] Sözen S Biri H Sinik Z Küpeli B Alkibay T Bozkirli I Comparison of the nuclear matrix protein 22 with voided urine cytology and BTA stat test in the diagnosis of transitional cell carcinoma of the bladder Eur Urol 1999 36 3 225 9 10.1159/000068002 10450007

[B9] Atsü N Ekici S Oge OO Ergen A Hasçelik G Ozen H False-positive results of the NMP22 test due to hematuria J Urol 2002 167 2 Pt 1 555 8 11792917 10.1016/S0022-5347(01)69084-0

[B10] Miyake M Goodison S Giacoia EG Rizwani W Ross S Rosser CJ Influencing factors on the NMP-22 urine assay: an experimental model BMC Urol 2012 12 23 10.1186/1471-2490-12-23 10.1186/1471-2490-12-23 22928931 PMC3480828

[B11] Rosser CJ Liu L Sun Y Villicana P McCullers M Bladder cancer-associated gene expression signatures identified by profiling of exfoliated urothelia Cancer Epidemiol Biomarkers Prev 2009 18 444 453 10.1158/1055-9965.EPI-08-1002 19190164 PMC2729268

[B12] Uriquidi V Goodison S Cai Y Sun Y Rosser CJ Molecular Biomarker Signature for the Non-Invasive Detection of Bladder Cancer 2012 CEBP 10.1158/1055-9965.EPI-12-0428 PMC3537330 23097579

[B13] Kreunin P Zhao J Rosser C Urquidi V Lubman DM Goodison S Bladder cancer associated glycoprotein signatures revealed by urinary proteomic profiling J Proteome Res 2007 6 7 2631 2639 Epub 2007 May 23 10.1021/pr0700807 17518487 PMC2668245

[B14] Yang N Feng S Shedden K Xie X Liu Y Urinary glycoprotein biomarker discovery for bladder cancer detection using LC-MS/MS and label-free quantification Clin Cancer Res 2011 17 3349 3359 10.1158/1078-0432.CCR-10-3121 21459797 PMC3096687

[B15] Urquidi V Kim J Chang M Dai Y Rosser CJ CCL18 In a multiplex urine-based assay for the detection of bladder cancer PLoS One 2012 7 e37797 10.1371/journal.pone.0037797 22629457 PMC3357344

[B16] Urquidi V Goodison S Kim J Chang M Dai Y VEGF, CA9 and angiogenin as a urinary biomarker for bladder cancer detection Urology 2012 79 1185 1188 22386755 10.1016/j.urology.2012.01.016 PMC3341520

[B17] Urquidi V Chang M Dai Y Kim J Wolfson ED L-8 as a urinary biomarker for the detection of bladder cancer BMC Urol 2012 12 12 15 10.1186/1471-2490-12-12 22559832 PMC3404900

[B18] Urquidi V Goodison S Ross Chang M Dai Y Diagnostic potential of urinary alpha 1-antitrypsin and apolipoprotein E in the detection of bladder cancer J Urol 2012 188 2211 2214 10.1016/j.juro.2012.08.232 23088986 PMC4013779

[B19] Goodison S Chang M Dai Y Urquidi V Rosser CJ A multi-analyte assay for the non-invasive detection of bladder cancer PLoS One 2012 7 10 e47469 doi:10.1371/journal.pone.0047469. Epub 2012 Oct 19 10.1371/journal.pone.0047469 23094052 PMC3477150

[B20] Bossuyt PM Reitsma JB Bruns DE Gatsonis CA Glasziou PP Towards complete and accurate reporting of studies of diagnostic accuracy: the STARD initiative Fam Pract 2004 21 4 10 10.1093/fampra/cmh103 14760036

[B21] Pisitkun T Johnstone R Knepper MA Discovery of urinary biomarkers Mol Cell Proteomics 2006 5 1760 71 10.1074/mcp.R600004-MCP200 16837576

[B22] Watanabe T Shinohara N Sazawa A Harabayashi T Ogiso Y Koyanagi T Takiguchi M Hashimoto A Kuzumaki N Yamashita M Tanaka M Grossman HB Benedict WF An improved intravesical model using human bladder cancer cell lines to optimize gene and other therapies Cancer Gene Ther 2000 7 12 1575 80 10.1038/sj.cgt.7700261 11228536

[B23] Rossi MR Masters JR Park S Todd JH Garrett SH Sens MA Somji S Nath J Sens DA The immortalized UROtsa cell line as a potential cell culture model of human urothelium Environ Health Perspect 2001 109 8 801 8 10.1289/ehp.01109801 11564615 PMC1240407

[B24] Fluss R Faraggi D Reiser B Estimation of the Youden Index and its associated cutoff point Biom J 2005 47 458 472 10.1002/bimj.200410135 16161804

[B25] Pepe MS Feng Z Janes H Bossuyt PM Potter JD Pivotal evaluation of the accuracy of a biomarker used for classification or prediction: standards for study design J Natl Cancer Inst 2008 100 1432 8 10.1093/jnci/djn326 18840817 PMC2567415

[B26] Chen J Yao Y Gong C Yu F Su S Liu B CCL18 From tumor-associated macrophages promotes breast cancer metastasis via PITPNM3 Cancer Cell 2011 19 541 55 10.1016/j.ccr.2011.02.006 21481794 PMC3107500

[B27] Zohny SF Fayed ST Clinical utility of circulating matrix metalloproteinase-7 (MMP-7), CC chemokine ligand 18 (CCL18) and CC chemokine ligand 11 (CCL11) as markers for diagnosis of epithelial ovarian cancer Med Oncol 2010 27 1246 53 10.1007/s12032-009-9366-x 19937162

[B28] Wang Q Li D Zhang W Tang B Li QQ Li L Evaluation of proteomics-identified CCL18 and CXCL1 as circulating tumor markers for differential diagnosis between ovarian carcinomas and benign pelvic masses 2011 Int: J Biol Markers 10.5301/JBM.2011.8616 21928244

[B29] Normandin K Peant B Le Page C Protease inhibitor SERPINA1 expression in epithelial ovarian cancer Clin Exp Metastasis 2010 27 55 10.1007/s10585-009-9303-6 20049513

[B30] Lindor NM Yang P Evans I Alpha-1-antitrypsin deficiency and smoking as risk factors for mismatch repair deficient colorectal cancer: a study from the colon cancer family registry Mol Genet Metab 2010 99 157 10.1016/j.ymgme.2009.09.010 19853488 PMC2818220

[B31] Hamrita B Chahed K Trimeche M Proteomics-based identification of alpha1-antitrypsin and haptoglobin precursors as novel serum markers in infiltrating ductal breast carcinomas Clin Chim Acta 2009 404 111 10.1016/j.cca.2009.03.033 19306859

[B32] Navarro-Muñoz M Ibernon M Bonet J Pérez V Pastor MC Bayés B Casado-Vela J Navarro M Ara J Espinal A Fluvià L Serra A López D Romero R Uromodulin and α(1)-antitrypsin urinary peptide analysis to differentiate glomerular kidney diseases Kidney Blood Press Res 2012 35 5 314 25 10.1159/000335383 22399069

[B33] Trojan B Tang A Chandrapal J Filleur S Nelius T The clinical usefulness of nuclear matrix protein-22 in patients with end-stage renal disease and microscopic hematuria Ren Fail 2013 35 1 72 6 10.3109/0886022X.2012.741648 23151051

[B34] Todenhöfer T Hennenlotter J Witstruk M Gakis G Aufderklamm S Kuehs U Stenzl A Schwentner C Influence of renal excretory function on the performance of urine based markers to detect bladder cancer J Urol 2012 187 1 68 73 10.1016/j.juro.2011.09.023 22088333

