# Supplementary figures and images for: Investigation of CCL18 and A1AT as potential urinary biomarkers for bladder cancer detection
Source: BMC Urol. 2013 Sep 5;13:42. doi: 10.1186/1471-2490-13-42 (PMC3846766; doi:10.1186/1471-2490-13-42)

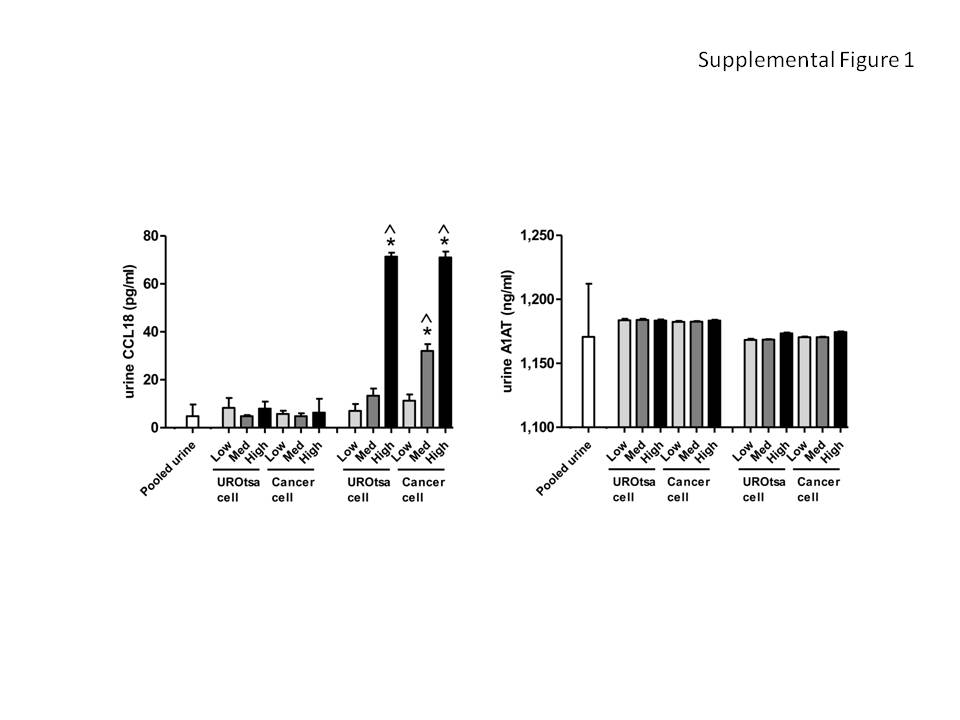

Supplement: Additional file 1 — Analysis of CCL18 and A1AT biomarker performance in an experimental model. Using the experimental model depicted in Figure 1, urinary levels of CCL18 and A1AT were analyzed by ELISA. The addition of high concentration of benign cell lysate or medium to high concentration of cancer cell lysate resulted in an increase in CCL18. The addition of cells or cell lysate did not alter A1AT levels. Error bars indicate standard deviations. *, significance (p < 0.05) compared to pooled urines from healthy subjects. ^, significance (p < 0.05) compared to corresponding lower concentration. [file 1471-2490-13-42-S1.jpeg]
